# Krüppel-like factor 4 control of immune cell function

**DOI:** 10.3389/fimmu.2025.1597210

**Published:** 2025-08-04

**Authors:** Tapatee Das, Elaine Wang, Yitian Xu, Harrison Yang, Xudong Liao, Mukesh K. Jain

**Affiliations:** ^1^ Department of Molecular Biology, Cell Biology & Biochemistry, Division of Biology and Medicine, Brown University, Providence, RI, United States; ^2^ The Warren Alpert Medical School, Brown University, Providence, RI, United States

**Keywords:** KLF4, immune cells, innate & adaptive immune response, SP family, homeostasis

## Abstract

Krüppel-like factor 4 (KLF4) belongs to a family of transcription factors that contain conserved zinc finger DNA binding domains, including specificity proteins (SPs) and Krüppel-like factors (KLFs). KLF4 plays a vital role in regulating cellular differentiation, proliferation and adaptation to a broad spectrum of internal and external cues. In the context of the immunity, KLF4 is appreciated as critical to both the innate and adaptive arms of the immune system. The current review article focuses on these aspects of KLF4 action as well as implications of this work for impacting human health.

## Highlights

- KLF4 acts as a negative regulator in both T and B cell proliferation, capable of arresting the progression of the cell cycle to maintain T and B cell quiescence.
- KLF4 regulates macrophage polarization and monocyte differentiation.
- KLF4 acts as a novel transcriptional regulator of neutrophil activation.

## 1 Introduction

Krüppel-like factors (Klfs) are a family of zinc-finger proteins (ZNF), contains three highly conserved C2H2 zinc-finger motifs that facilitate specific DNA binding, enabling it to act as both a transcription activator and repressor. A key feature of the Klf family is that it contains 3 Cys2/His2 ZNF. Zinc fingers 1 and 2 have 23 amino acids, while finger 3 has only 21 amino acids while finger 3 has only 21 amino acids (1). The Kruppel-Like Factor family of regulatory proteins having 18 members of transcription factors. KLF proteins bind to specific DNA sequences to either activate or repress the transcription of target gene, expression and cellular functions (2) including differentiation, proliferation, and homeostasis across major physiologic systems. In particular, work to date supports a critical role in the endocrine, muscular (smooth and striated), nervous, cardiovascular and immune systems. Within different immune cell types like T cells, B cells and myeloid cells, Klfs essentially act as key regulators by controlling gene expression depending on the specific Klfs involved (3). Different Klf family members can have distinct roles in immune cells with some promoting immune responses while others acting as suppressors depending on the cellular context and environmental cues. KlFs can influence the expression of inflammatory cytokines and chemokines impacting the inflammatory response in different immune cell types (4). Aberrant KLF expression has been linked to various immune related diseases like autoimmune disorders, cancer, and chronic inflammatory conditions. KLFs can both promote and inhibit cell proliferation, depending on the specific KLF and cellular context. For instance, KLF5 promotes cell proliferation, while KLF4 inhibits it. Similarly, KLFs play vital roles in cell differentiation, such as KLF1 in erythroid differentiation and KLF4 in goblet cell differentiation. Some KLFs, like KLF4, are involved in the regulation of apoptosis, sometimes acting as tumour suppressors by promoting apoptosis, while in other instances exhibiting anti-apoptotic effects. KLFs are critical regulators during development, influencing processes like erythropoiesis, adipogenesis, and skeletal development.

Certain KLFs, like KLF2 and KLF4, are involved in regulating immune responses, influencing leukocyte development and function. They are also implicated in inflammatory diseases.

KLFs related to the immune system are Klf1,Klf2,Klf3,Klf4,Klf6 and Klf16. KLF2 is particularly well studied for its role in maintaining immune cell quiescence and regulating T cell activation while other KLFs like KLF5 and KLF14 are also implicated in immune cell function (1). Klf4 has important functions in the innate and adaptive immune system. The adaptive immune system is responsible for generating immunological response and immunological memory. Regulation of adaptive immunity including B cell and T cell biology was mainly understood from the protein and microRNA perspective. KLF4 directly regulates miR-182 cluster expression in human embryonic stem cells (hESCs) and in melanoma tumours, in which the miR-182 cluster is highly expressed and has a pro-metastatic role. Furthermore, higher KLF4 expression was found to be associated with metastatic progression and poor patient outcome. Loss of function experiments revealed that KLF4 is required for melanoma cell maintenance. These findings provide new insights into the regulation of the miR-182 cluster expression and new opportunities for therapeutic intervention in tumors in which the KLF4-miR-182 cluster axis is deregulated (5). However, long non-coding RNAs (lncRNAs) are an emerging class of non-coding RNAs (ncRNAs) that influence key factors in lymphocyte biology such as NOTCH, PAX5, MYC and EZH2. LncRNAs were described to modulate lymphocyte activation by regulating pathways such as NFAT, NFκB, MYC, interferon and TCR/BCR signalling (*NRON, NKILA, BCALM, GAS5, PVT1*), and cell effector functions (*IFNG-AS1, TH2-LCR*) (6).

**Figure 1:**
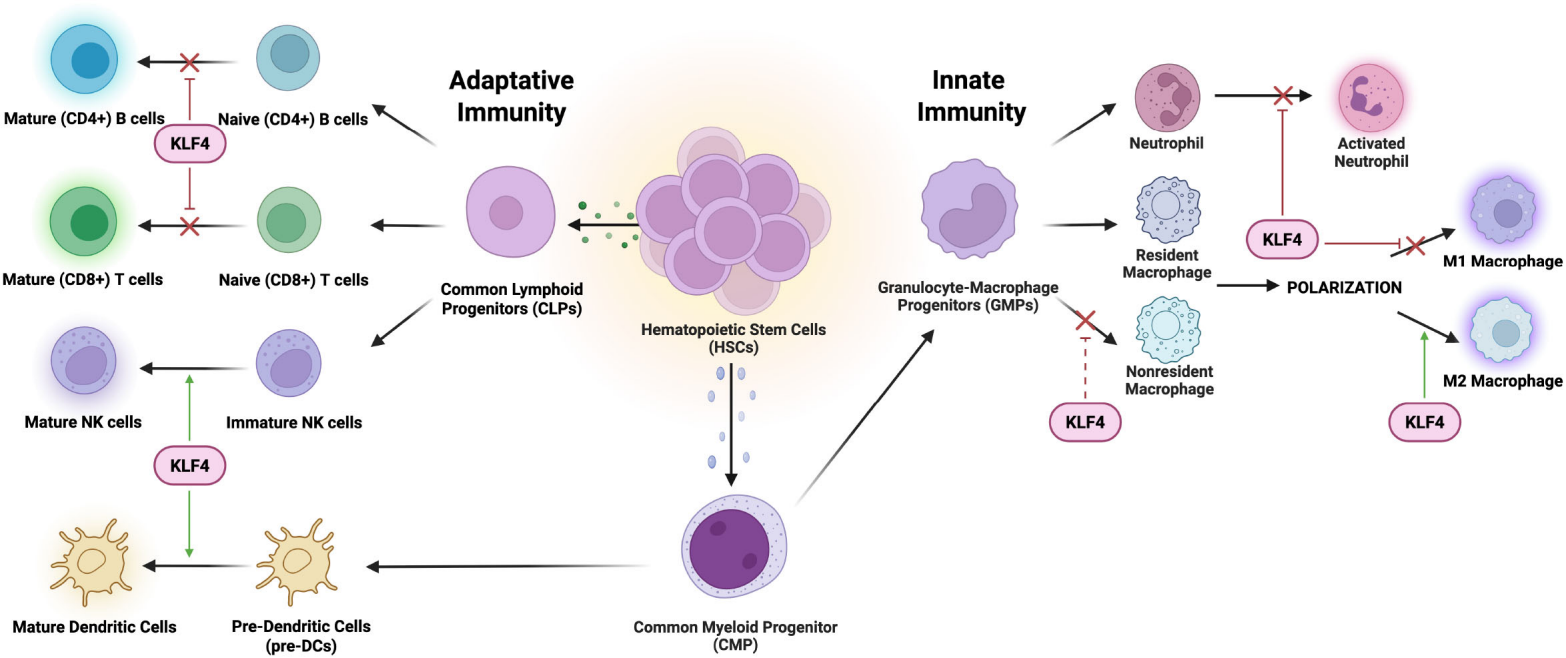
Interaction of KLF4 with innate and adaptive immune cells. KLF4 plays a crucial role in regulating the differentiation and function of various immune cells. In an innate immunity context, KLF4 promotes anti-inflammatory responses influencing tissue repair and immune resolution. In an adaptive immunity context, KLF4 is involved in immune modulation, specifically impacting activation, proliferation, and cytokine production to maintain immune homeostasis.

This review focuses on the crucial role of KLF4 in maintaining immune cell system’s balance particularly in regulating immune responses and function of various immune cells (**Figure 1**).

## 2 The role of KLF4 in monocyte and macrophage biology

The innate immune system functions as the body’s first line of defence through the initiation of a nonspecific rapid response to pathogens and infections, while also serving a critical role in the overall regulation of tissue homeostasis and immunity surveillance (7). Myeloid lineage-enriched transcription factors, drive the commitment of progenitor cells toward a stage-specific monocyte-macrophage differentiation program (8).

### 2.1 Monocyte-macrophage introduction

The innate immune system functions as the body’s first line of defence through the initiation of a nonspecific rapid response to pathogens and infections, while also serving a critical role in the overall regulation of tissue homeostasis and immunity surveillance (7). Monocytes, a subset of white blood cells originating from hematopoietic stem cells (HSCs) in the bone marrow, patrol the bloodstream for sites of inflammation and, upon migration to tissues, differentiate into their tissue-specific macrophages (9). Once relocated into the tissue, these differentiated and highly plastic macrophages undergo polarization in adaptation to their specific microenvironment, a process tightly regulated under the KLF4 transcriptional control (8, 10).

In principle, there are two subsets of macrophages, namely tissue resident and non-resident macrophages. Resident macrophages (Ly6C^lo^/CCR2^-^), originating from yolk sac-derived erythro-myeloid progenitors, predominantly exhibit an M2-like anti-inflammatory phenotype under homeostatic conditions, contributing antagonistic roles of inflammation, such as mitigating insulin resistance, eliminating parasites, and promoting tissue remodelling and repair (11–13). In contrast, non-resident (blood-borne infiltrating) macrophages, derived from circulating (Ly6C^hi^) monocytes recruited by the CCL2-CCR2 chemotaxis pathway, are more likely to adopt an M1-like proinflammatory phenotype to enhance pathogen clearance and increase blood vessel permeability and the activation of other inflammatory mediators (14–17). Recent studies have established the crucial role of KLF4 in modulating macrophage polarization, influencing the balance between proinflammatory M1 and anti-inflammatory M2 phenotypes, thus contributing to immune homeostasis and the resolution of inflammation (1).

### 2.2 Regulation of monocyte differentiation

Monocyte differentiation is embedded within the broader framework of haematopoiesis – a highly transcriptionally regulated process that drives the specialization of HSCs into various blood lineages. HSCs first give rise to common myeloid progenitors (CMPs), which differentiate into granulocyte-macrophage progenitors (GMPs). GMPs can then commit to either granulocytic or monocytic lineages depending on specific transcription factor cues (18).

**Figure 2:**
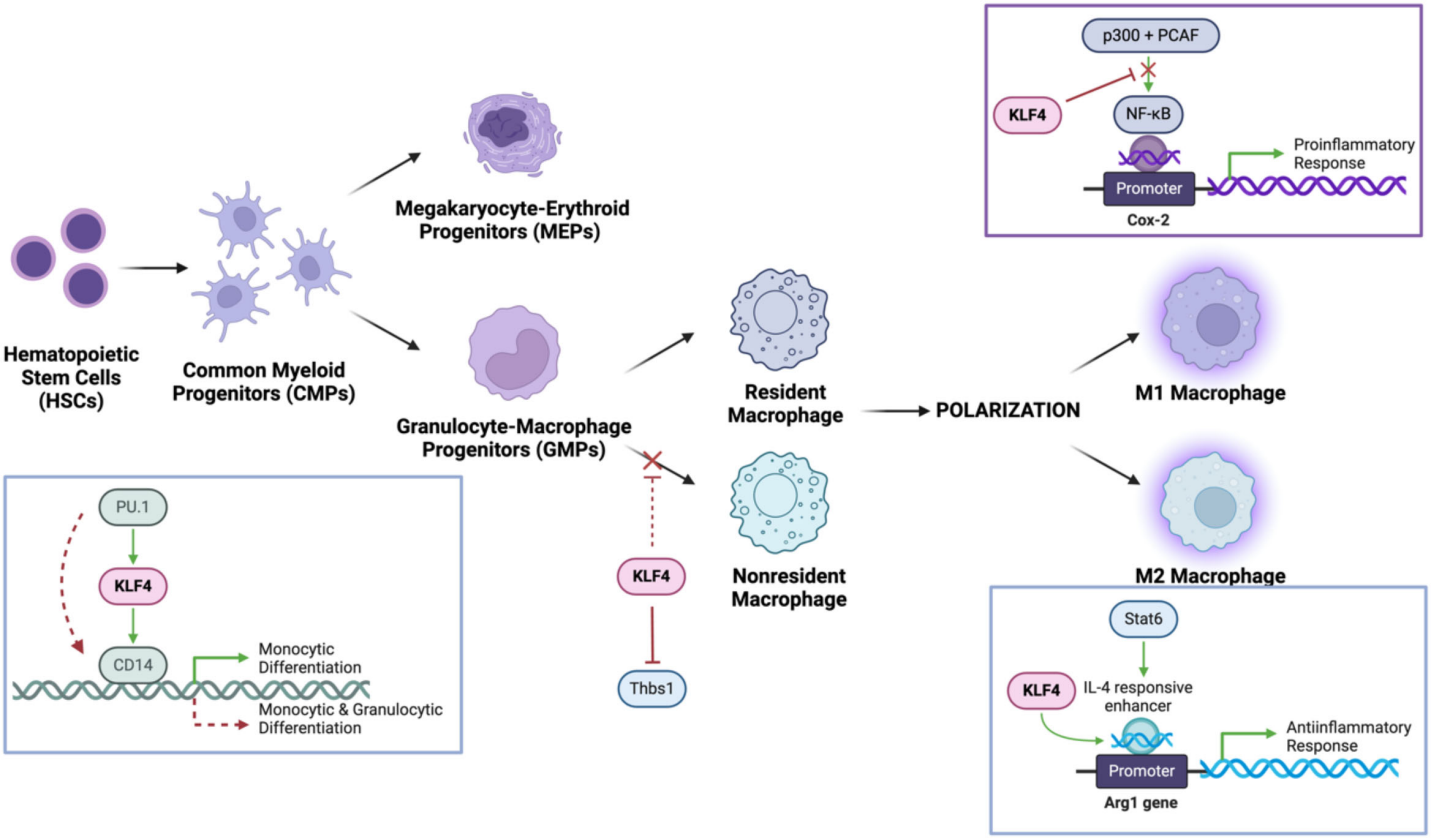
KLF4 mechanistically modulates monocyte differentiation and macrophage populations and polarizations. It cooperates with PU.1 to promote monocyte differentiation while upregulating CD14, a key monocyte marker. In macrophages, KLF4 inhibits NF-κB signalling, reducing proinflammatory M1 polarization, while enhancing Stat6/IL-4 signalling to drive M2 macrophage polarization, facilitating tissue repair and anti-inflammatory responses.

The myeloid differentiation process is modulated by the induction of lineage-restricted transcription factors such as PU.1, a member of the ETS family of transcription factors, that bind to consensus GGAA-like motifs, for GMPs differentiation and GATA-1 as an antagonist to drive megakaryocyte-erythroid progenitors (MEPs) differentiation (19–21). KLF4, a downstream target gene of PU.1, binds the CD14 promoter, thus specifying monocyte commitment. In PU.1-deficient mouse fetal liver cells, KLF4 overexpression restores monocyte differentiation in the absence of PU.1, supporting a partially compensatory or synergistic relationship between the two transcription factors (22) (**Figure 2**). Thus, KLF4 acts as both a determinant and effector of monocyte lineage commitment and differentiation. The expression of KLF4 is present in monocyte-lineage cell lines (e.g., THP-1, U-937) but absent in other hematopoietic cell types, underscoring its specificity.

Functionally, KLF4 not only induces monocytic surface markers (CD11b, CD14; while not impacting granulocytic and lymphocytic markers) but also causes morphological changes (23). These characteristic changes in cellular morphology, include increased cytoplasmic size, smaller and more condensed nuclei, and ruffled cell edges, consistent with the acquisition of a monocytic phenotype (7, 24). Understanding these regulatory mechanisms not only advances our knowledge of hematopoietic lineage commitment but also provides potential targets for therapeutic modulation in inflammatory and myeloid-related disorders.

### 2.3 Transcriptional control of macrophage polarization

Macrophages can undergo polarization in adaption to their specific microenvironment, a process tightly regulated by KLF4 (10). The heterogeneous cell population of macrophages plays an essential role in the innate immune response due to their remarkable plasticity, allowing them to adopt distinctive functional phenotypes (M1/M2) in response to tissue environment cues governing polarization (25, 26). This binary classification of macrophages is an oversimplification of a more dynamic spectrum of activation states. Given its regulatory role, KLF4 serves as a key transcription factor essential in modulating and regulating macrophage polarization, to facilitate tissue repair but also suppresses excessive inflammation, highlighting its dual role in immune homeostasis (27).

#### 2.3.1 KLF4 promotes M2 macrophage polarization

The anti-inflammatory M2 macrophage primarily functions to remodel and organize components of the extracellular matrix to facilitate the clearance of cellular debris (via matrix metalloproteinases [MMPs]) and secretion of TFG-β to promote tissue repair (28). This KLF4-promoted M2 macrophage polarization is modulated by cytokines, IL-4 and IL-13 to achieve optimal expression of target genes (arginase-1, mannose receptor, resistin-like α, and chitinase 3-like 3) expressing anti-inflammatory markers (**Figure 2**). Essential to M2 activity, arginase 1 (encoded within the Arg1 gene) is an enzyme that degrades L-arginine, thereby limiting nitric oxide (NO) production while promoting polyamine synthesis (29). The well-characterized Arg1 promoter is mechanically regulated by KLF4 determined by the consensus KLF-binding sites (CACCC) located in the IL-4-responsive enhancer region tangentially to the Stat6-binding site. Stat6, as another transcription factor involved in regulating immune homeostasis and M2 polarization, cooperates with KLF4 to induced M2 genes in response to IL-4 activation. To elucidate the manifestation of an optimal KLF4 cooperative activity, Stat6-null macrophages observed decreased recruitment of KLF4 to Arg1 enhancer region following IL-4 treatment, indicative of a dependent relationship to Stat6 and synergistic activation requires intact KLF4 and Stat6 factors (27). Overexpression of Arg1 can result in excessive tissue repair and scarring (fibrosis), while insufficient Arg1 activity can impair healing processes. Interestingly, overexpression of KLF4 resulted in the upregulation of the PPARγ (Peroxisome proliferator-activated receptor gamma) to suppress M1-associated proinflammatory cytokines via inhibition of NF-κB pathway while simultaneously enhancing IL-4/Stat6 signalling. Cooperative interactions with PPARγ and Stat6 establish KLF4 as a critical mediator of M2 macrophage polarization (30).

#### 2.3.2 KLF4 inhibits M1 macrophage polarization

The M1 macrophage phenotype adopts a proinflammatory role via the stimulation of macrophages with bacterial endotoxin lipopolysaccharide (LPS), characterized by the increased production of antimicrobial effector molecules such as prostaglandins (via induction of prostaglandin-endoperoxide synthase 2 [Cox-2]), nitric oxide (via inducible nitric oxide synthase [iNOS]), and proinflammatory cytokines (TNF-α, IL-1β) (31, 32). Interestingly, M2 macrophages can be reprogrammed to M1-like macrophages due to the downregulation of their characteristic anti-inflammatory markers under proinflammatory signals like LPS or INF-γ via the activation of the JAK/STAT pathway (33, 34). As the primary classic proinflammatory target, the Cox-2 promoter doesn’t have a canonical KLF-binding site, however, studies of KLF4-deficient macrophages displayed significantly enhanced recruitment of histone acetyltransferases (p300 and PCAF) to activate the transcription of proinflammatory gene promoters specific to the NF-κB pathway (35, 36) (**Figure 2**). Amplified bactericidal activity is observed in KLF4-deficient M1 polarization, as the production of ROS-generating enzyme NADPH oxidase 1 (Nox1), Infg, and Tnfa was in response to both Gram-negative and Gram-positive bacteria (27). Further studies of myeloid KLF4 deficiency mice exhibited delayed wound closing measured by upregulated expression of iNOS and TNF-α, determined by increased levels of tissue destruction correlated to promoted insulin resistance (37–39). With the NF-κB signalling pathway serving as a key regulator of M1 polarization, KLF4 may act as a negative regulator, suppressing excessive inflammation and rampant bactericidal activity while facilitating proper wound healing.

## 3 KLF4 and its role in neutrophils

Neutrophils form the largest component of circulating white blood cells and serve as the first line of immune defence against microbial infections (40). These motile immune cells are recruited to sites of infection or injury, where they eliminate pathogens through mechanisms such as phagocytosis, degranulation, and the release of neutrophil extracellular traps (NETs) (41). While their primary role is to maintain host defence, neutrophils can also contribute to tissue damage and chronic inflammation when their activation is dysregulated, leading to autoimmune diseases like rheumatoid arthritis, phospholipid antibody syndrome, or systemic lupus erythematosus (SLE) (42). As such, it is crucial to understand the molecular pathways governing neutrophil function and activity. To date, KLF4 has emerged as a crucial player in modulating neutrophil activation and function in the context of acute infection and chronic inflammation. It balances pro-inflammatory responses necessary for pathogen clearance with mechanisms that prevent excessive inflammation upholding a dual role in host defence and tissue homeostasis.

### 3.1 KLF4-deficiency on neutrophil granule proteins and cytokines

Neutrophil granules are membrane-bound vesicles storing pro- and anti-inflammatory molecules, that are released through a process termed degranulation to execute pathogen elimination and tissue remodelling functions (43). Among these molecules are essential antimicrobial and cytotoxic molecules, such as matrix metalloproteinase-9 (MMP-9) and myeloperoxidase (MPO) (44). In wild-type neutrophils, lipopolysaccharide (LPS) stimulation upregulates MMP-9 mRNA, promoting granule release. However, KLF4-deficient neutrophils exhibit a significant reduction in MMP-9 mRNA levels, indicating that KLF4 is necessary for proper transcriptional regulation of granule-associated proteins. Furthermore, reduced secretion of MMP-9 and MPO in these neutrophils suggests that KLF4 deficiency primarily disrupts granule content; since MMP-9 mRNA levels were already suppressed in KLF4-deficient neutrophils, this deficiency in secretion is not due to impaired degranulation but rather to a reduction or defect in granule content (45).

Beyond granule-associated proteins, KLF4-deficient neutrophils also exhibit altered cytokine secretion. Proinflammatory cytokines such as TNF-α, keratinocyte chemoattractant (KC), and IL-1β, along with the anti-inflammatory cytokine IL-10, coordinate the innate immune response (46). Upon *Streptococcus pneumoniae* stimulation, KLF-deficient neutrophils showed significantly reduced TNF-α and KC secretion, while IL-10 release was elevated compared to the wild-type control. IL-1β levels remained unaffected, possibly suggesting that KLF4 selectively regulated specific cytokine pathways in neutrophils. Notably, these changes were observed in blood-derived PMNs but not in remaining white blood cells (WBCΔPMNs), indicating potential differences in KLF4-dependent cytokine regulation amongst neutrophil populations (47). Together, these findings highlight KLF4’s essential role in promoting proinflammatory cytokine release while restraining anti-inflammatory signalling, making it a critical regulator of neutrophil-mediated immune responses.

### 3.2 Neutrophil KLF4-deficiency and susceptibility to bacterial infection

Effective bacterial clearance mechanisms are essential for neutrophils to maintain host defence from bacterial infections (48). The loss of KLF4 disrupts these processes, compromising immune responses and increasing susceptibility to infection. Following intraperitoneal *E. coli* infection, myeloid-specific KLF4-deficient mice exhibited significantly higher mortality rates than controls. Increased bacterial burden in these mice points to uncontrolled infection and subsequent development of bacteraemia. Furthermore, circulating levels of TNF-α, MPO, and MMP-9 were significantly lower in myeloid-specific KLF4-deficient mice, signifying a diminished host defence mechanism. Impaired bacterial killing capabilities in KLF4-deficient neutrophils ex vivo further support this observation (45). KLF4-deficient murine polymorphonuclear neutrophils (PMNs) also exhibit reduced pneumococcal killing. After incubation with opsonized *S. pneumoniae* D39 or R6× for three hours, KLF4-deficient blood-derived PMNs showed significantly reduced bacterial clearance (47). Although the precise mechanism by which KLF4 regulates neutrophil antimicrobial function remains unclear, these findings suggest that KLF4 plays a central role in coordinating the transcriptional response required for effective bacterial killing (49).

### 3.3 Inflammatory response in KLF4-deficient neutrophils

Neutrophil function is protective in bacterial killing, but excessive systemic inflammation can lead to septic shock and death (50). Interestingly, while KLF4 deficiency impairs the immune response to bacterial infection, it appears to confer resistance to excessive inflammation in response to direct challenge with endotoxin (45). LPS-induced mortality rate is greatly reduced in myeloid-specific KLF4-deficient mice compared with the control, along with lower levels of pro-inflammatory factors MPO and TNF-α. Beyond acute infection, neutrophils also play a key role in chronic inflammatory diseases, where their interactions with other immune cells influence disease progression (51). Specifically, neutrophils may influence chronic inflammation in the experimental autoimmune encephalomyelitis (EAE) model, which mimics multiple sclerosis (MS) in humans (52, 53). KLF4-deficient neutrophils impaired chronic inflammation, exhibited delayed disease onset, and significantly reduced EAE severity compared to wild-type controls despite a 100% incidence. At the disease onset phase, a significant reduction in CNS-infiltrating neutrophils, T helper cells, B cells, and dendritic cells was observed in myeloid-specific KLF4-deficient mice, further suggesting that KLF4-deficient neutrophils are defective in mediating chronic inflammation (45). These findings reinforce the role of KLF4 in promoting inflammatory responses, with its absence reducing acute endotoxin-induced inflammation and chronic autoimmune-mediated neuroinflammation.

### 3.4 Mechanisms of KLF4 regulation in neutrophils

**Figure 3:**
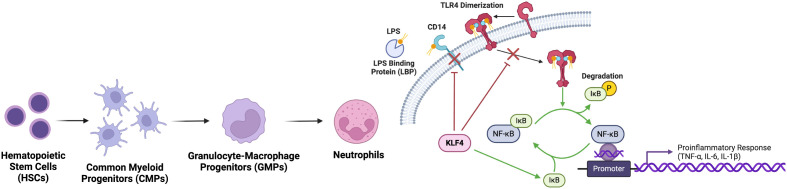
KLF4 inhibits neutrophil activation of proinflammatory response. Via the suppression of NF-κB pathway, nuclear translocation and subsequent transcription of proinflammatory genes is prevented. Upstream, KLF4 downregulates TLR4 and CD14, reducing neutrophil responsiveness to bacterial LPS and dampening the initiation of inflammatory cascades, thereby limiting excessive inflammation.

Previously, TLR4 activation by LPS has been shown to induce KLF4 expression in murine neutrophils (45). KLF4-deficient neutrophils exhibited impaired responses to LPS stimulation, which typically activates TLR4 and initiates a signalling cascade leading to the activation of the IкB kinase (IKK) complex. This complex phosphorylates IкBα, resulting in its degradation and the subsequent release of NF-кB, a key transcription factor of pro-inflammatory genes (54). In KLF4-deficient neutrophils, LPS-induced phosphorylation and degradation of IкBa are significantly attenuated, resulting in reduced NF-кB activation and decreased transcription of its target genes such as TNF-α (45). One of the key mechanisms by which KLF4 influences this pathway is through the regulation of CD14, a crucial co-receptor for TLR4 (55). CD14 aids TLR4 in recognition of bacterial components like LPS, and typically, neutrophils upregulate CD14 levels upon encountering LPS. However, in KLF4-deficient neutrophils, both CD14 mRNA and surface protein expression were reduced, both at baseline and after LPS activation. This reduction in CD14 expression impairs the ability of neutrophils to efficiently recognize bacteria stimuli, weakening the activation of the downstream TLR4-NF-кB signalling cascade. While other components of the TLR4 pathway remain unaffected by KLF4 deficiency, the decreased expression of CD14 is a critical factor in the reduced responsiveness to LPS in neutrophils (45) (**Figure 3**).

Other findings testing various TLR pathways (TLR2, TLR4, and TLR9) demonstrated that activation of a single TLR by its respective agonist (MALP-2, LPS, or CpG) was insufficient to induce KLF4 expression in neutrophils, macrophages, or epithelial cells (47). Possible explanations for the difference in findings is that mouse and human neutrophils respond to LPS differently from variations in TLR4 or the use of different types of LPS from different bacterial sources. Additionally, neutrophils do not always respond strongly to a single stimulus but can become more reactive after priming (56). This suggests that multiple stimuli might be needed to induce KLF4 expression, which was observed with *S. pneumoniae* stimulation (47).

## 4 KLF4 in T and B lymphocytes

T and B lymphocytes are white blood cells that play a significant role in the adaptive immune response to infection. During the immune response, naïve T cells are activated by certain antigens, causing them to proliferate and differentiate into mature T cells, which are then recruited to different sites of infection (57). On the other hand, the primary function of B cells is to release antibodies, which they do by differentiating into plasmocytes upon encountering certain antigens (58). Despite serving critical roles in the immune response, T and B cells are also predecessors of destructive cancers: T cell acute lymphoblastic leukaemia (T ALL), which can arise from malignant thymocytes in certain stages of T cell differentiation, and Hodgkin’s Lymphoma, derived from mutations in the germinal centres of B cells. As such, it is critical to elucidate the molecular mechanisms regulating the differentiation and development of T and B lymphocytes.

T cell quiescence is regulated by the transcription factor KLF4, as well as different FOXO proteins. The FOXO proteins are a family of transcription factors known for regulating key homeostatic processes, including cell proliferation (59). It has been suggested that KLF4 serves a regulatory role in the differentiation of CD8+ T cells, while also inhibiting the proliferation of B cells downstream of certain FOXO proteins (60). However, the exact role of KLF4 in developing T and B cells has not yet been clarified.

### 4.1 KLF4 in regulation of CD8+ T cell development

**Figure 4:**
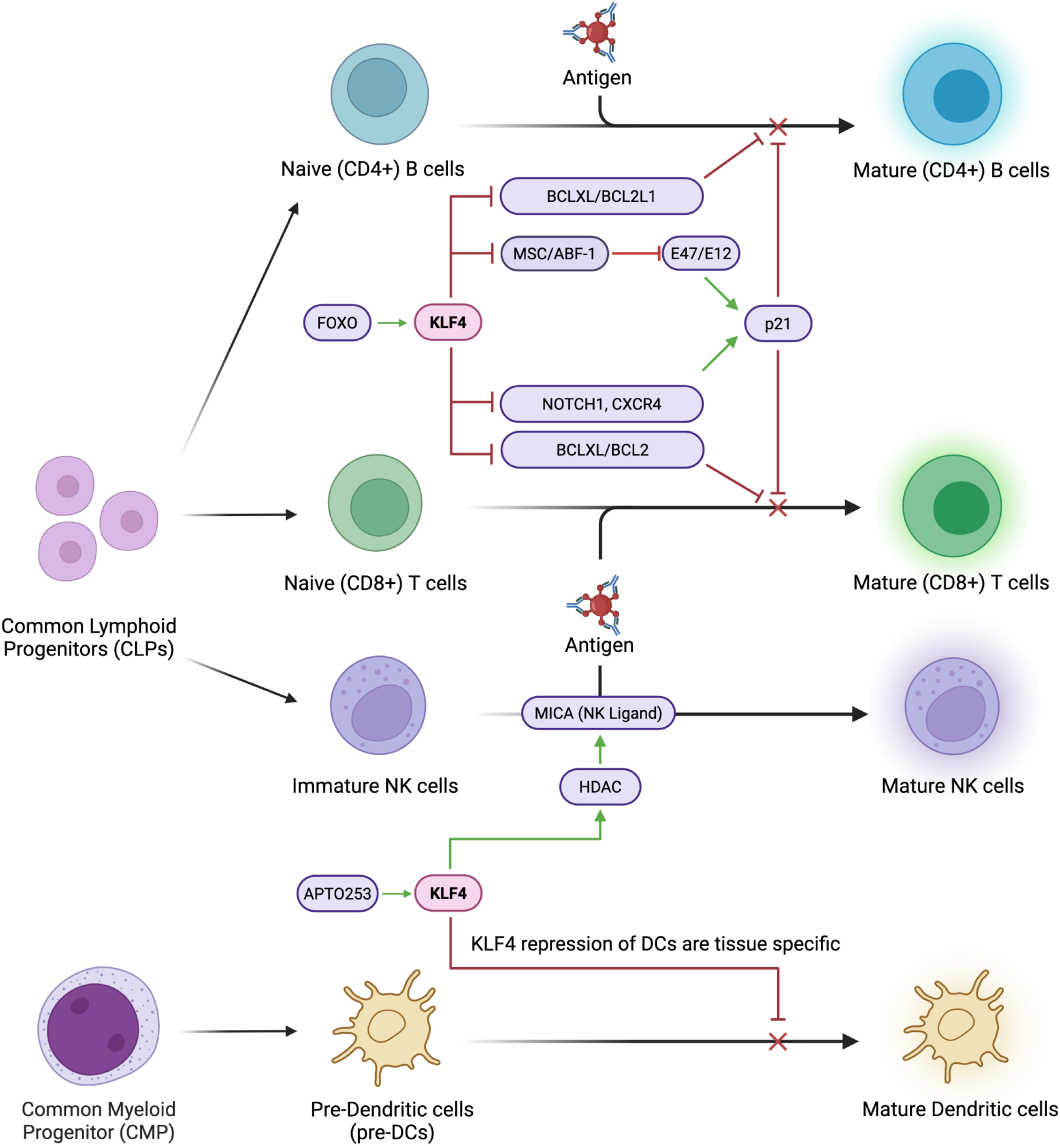
KLF4 systematically inhibits lymphocyte maturation. KLF4 systematically inhibits lymphocyte maturation by repressing key regulatory and proliferation factors such as MSC/ABF1 and E47/E12. It downregulates NOTCH1, a critical driver of T cell commitment, and reduces CXCR4 expression, impairing lymphocyte migration and homing. Additionally, KLF4 suppresses anti-apoptotic genes like BCL2 and BCLXL, leading to increased susceptibility to apoptosis and impaired lymphocyte survival.

Functional CD8+ T cells, otherwise known as cytotoxic T cells or killer T cells, originate from naïve CD8+ T cells in the bone marrow and undergo a rapid expansion, differentiation, and selection in the thymus (61). Once matured, cytotoxic T cells become mono-specific CD8+ T cells capable of contributing to the immune response. Novel research indicates following enhanced T cell receptor (TCR) activation by TCR crosslink, naïve CD8+ T cells in Klf4-deficient mice demonstrated increased proliferation (60). In the same experiment, these Klf4-deficient naïve CD8+ T cells also increased in proliferation in response to bacterial infection – specifically, when upon infection with a strain of *Listeria monocytogenes-*OVA, Klf4-deficient naïve CD8+ T cells generated more memory CD8+ T cells in both the primary and secondary responses to the infection. Additionally, circulating CD8+ T cells remain quiescent when under KLF4 expression (62) maintained through activation of p21, a kinase inhibitor involved in cell cycle regulation (63). CD8+ T cells from Klf4-deficient mice upregulated proliferation following *in vitro* TCR crosslink activation and increased *in vivo* homeostatic population expansion in the spleen. The presence of KLF4 modulates repression of T cell development in T ALL by inducing apoptosis through suppression of the BCL2/BCLXL genes (**Figure 4**). Previous studies also reveal that the KLF4 gene becomes hypermethylated in cases of T ALL, while forced KLF4 overexpression suppresses its downstream signals, inhibiting T ALL progression and inducing T cell apoptosis (64). Mechanistically, through the binding of KLF4 to the promoters of T cell-associated genes, *NOTCH1*, *BCL2*, and *CXCR4*, repression and inhibition of T Cell proliferation is initiated (65). The results of these *in vitro* experiments posit KLF4 as a negative regulator in T cell-associated genes and, therefore, T cell proliferation. Specifically, KLF4 functions as a negative regulator in the proliferation of (naïve) CD8+ T cells under increased TCR activation/stimulation *in vitro*. Disruption of KLF4 activity enhances proliferation, and KLF4 also plays a key regulating role in the proliferation of functional memory CD8+ T cells.

### 4.2 KLF4 in B cell development

B Cells exist naturally in the quiescent state, non-dividing and inactive until they encounter the appropriate antigen. In active B cells, KLF4 becomes heavily downregulated, and *in vitro* studies indicate that an induced KLF4 expression prevents and decreases B cell cycle progression to the S phase via the following modulation of the known target genes of KLF4: increased p21 activity and decreased cyclin D2 and c-Myc activity (66). Forced expression of KLF4 also increased cell death in those proliferating B cells. However, the KLF4-deficient B cells displayed similar survival rates and expression as normal B cells, suggesting that the loss of KLF4 had no noticeable impact on B cell proliferation and development. It appears KLF4 serves a redundant role as other members of the KLF family, like KLF2/KLF3, which are also highly expressed in naïve B cells and downregulated upon activation, and loss of KLF4 can be compensated by other KLF members. KLF4’s function is reminiscent of different members of the FOXO protein family, like FOXO1 and FOXO3a, which induce cell cycle arrest to G1 and increase apoptosis upon forced expression (67). The FOXO protein family may be involved in KLF4 transcription, and KLF4’s regulation by FOXO proteins is a critical mechanism that determines B cell proliferation and survival.

In another study, KLF4 is shown to inhibit B cell proliferation in patients with B cell lymphomas, like Burkitt Lymphoma, follicular lymphoma, and classic Hodgkin lymphoma (68). Primary cases of B-cell lymphomas revealed that the KLF4 promoter was methylated, thus silencing the expression of KLF4 in the B cells (**Figure 4**). By overexpressing KLF4 in Burkitt lymphoma cell lines, researchers were able to induce cell cycle arrest in G_o_ and G_1_, pausing the proliferation of B cells. In Hodgkin’s lymphoma cell lines, KLF4 overexpression resulted in increased apoptosis through the activation of the BAK1 proapoptotic gene. BAK1 activity is sequestered by the MCL1 and BCL2L1/BCL_-XL_ pathways, and is only functional if it exists in higher concentrations than the sequestering capabilities of its regulators. KLF4 may be able to overstimulate BAK1 production, forcing it to become active and induce apoptosis in B cells. Interestingly, KLF4 also has a strong regulatory effect on the MSC/ABF-1 repressor, which is highly expressed in classic Hodgkin’s Lymphoma, follicular lymphoma, and Burkitt Lymphoma (69, 70). MSC/ABF-1 is a helix-loop-helix protein that suppresses the transactivating potential of E-box transcription factors, E47/E12 (71) which are downstream proteins involved in B cell proliferation. E47/E12 regulate proliferation by activating the p21 gene (72). It is plausible that KLF4 controls the proliferation of B cells by inhibiting the MSC/ABF-1 factor, which increases activity of E47/E12, which in turn activates the p21 protein and delays the cell cycle.

### 4.3 KLF4 in dendritic cells

During infection, DCs are responsible for initiating the adaptive immune response, activating T cells by capturing and presenting target antigens (73, 74). There are several subsets of DCs, like conventional DCs (cDCs), Langerhans cells (LCs), monocyte-derived DCs, among others, all having specific functions in response to infection/disease (75, 76) (**Figure 4**).

In DC differentiation, KLF4 expression positively correlates with the proinflammatory characteristics of that DC subset. KLF4 activity is induced in dermal DC and monocyte-derived DC differentiation, but is repressed to enable differentiation in LCs (77). Specifically, epithelial Notch signalling represses KLF4 in developing LCs, causing Runt-related transcription factor 3 derepression in response to TGF-β1, which ultimately enables differentiation into LCs due to low cytokine expression markers. KLF4 is also important in cDC development and function, particularly in IRF4-expressing cDCs that promote Th2 immune responses. *In vivo* cDCs with conditional deletion of KLF4 demonstrated impaired Th2 cell response to infection by *Schistosoma mansoni* and house dust mites, although Th1/Th17 cell responses were unaffected in response to other infections (78). In different tissues, KLF4 deletion decreased expression of IRF4 in pre-cDC subsets, and caused selective loss of cDC subsets expressing IRF4.

KLF4 activity also modulates inflammatory immune responses, including the production of inflammatory molecules like IL-6 by DCs. Rosenzweig et al. found that KLF4 plays dual functions to modulate expression of IL-6, directly activating the IL-6 promoter and remodelling chromatin (79). Further, they showed that DCs lacking KLF4 had significantly reduced levels of IL-6 mRNA and protein, although IL-6 was not fully absent.

### 4.4 KLF4 in natural killer cells

Natural Killer (NK) cells are fundamental cytokines in the innate immune response pathway to cancer (79), inhibiting proliferation and migration/colonization of distant tissues to combat primary tumour cells and metastasis (79). NK cells also produce large amounts of cytokines like interferon-γ, modulating adaptive immune responses and participating in similar pathways (80, 81).

KLF4 has been shown to promote survival of NK cells in the spleen, and also maintain the number of cDCs in the spleen (82) mutated the KLF4 gene in cre-transgenic mice, and found that somatic deletion leads to heavily reduced numbers of NK cells in the blood and spleen but not in bone marrow (BM), liver, or lymph nodes (82). Functional and immunophenotypic analyses suggested increased NK cell apoptosis in these cells, and that survival is dependent on BM-derived hematopoietic cells from the spleen. Further, numbers of CD11c^hi^ DCs, which promote NK cell survival, were significantly reduced in the KLF4-deficient mice, suggesting that the KLF4 gene is associated with the maintenance of spleen DCs, which support differentiation and survival of NK cells.

KLF4 additionally plays a role in upregulating NK cell ligands, specifically the NKG2D ligand *MICA*, which stimulates immune responses of NK cells (**Figure 4**). Alkhayer et al. determined that the *MICA* promoter contains KLF4-binding motifs, and that in acute myeloid leukaemia (AML), KLF4 mediates inducible expression of *MICA* (83). Upon inhibition/ablation of KLF4, HDAC-mediated upregulation of *MICA* is also reduced. Moreover, the APTO253 molecule, which is a known KLF4 inducer, was found to upregulate *MICA* in AML cells. APTO253-treated AML cells were also rendered more susceptible to termination by NK cells.

## 5 Regulatory function of KLF4 across major immune cell types with respect to diseases

**Figure 5:**
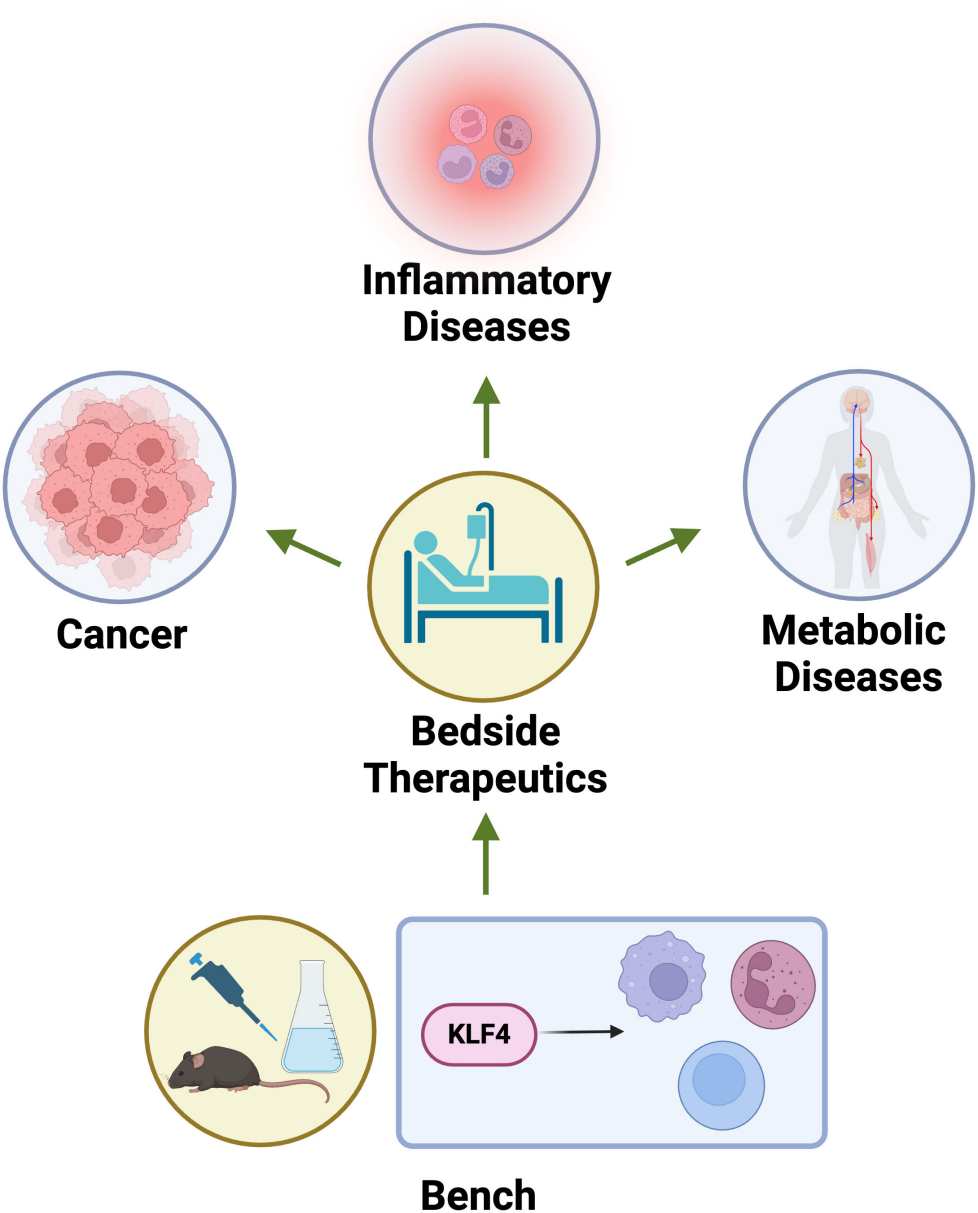
Bench to bedside applications of KLF4. KLF4 serves as critical therapeutic targets bridging bench to bedside applications in cancer, inflammatory diseases, and metabolic disorders. In cancer, modulating KLF4 expression can influence tumor suppression or progression depending on the context. In inflammatory diseases, KLF4’s role in macrophage polarization and NF-κB inhibition offers potential for controlling chronic inflammation. In metabolic diseases, KLF4 regulates lipid metabolism and insulin sensitivity, presenting a target for metabolic syndrome and diabetes therapeutics.

KLF4 plays a multifaceted role in the immune system, impacting various cell types and influencing disease progression. Its activity is context-dependent, leading to both pro- and anti-inflammatory effects depending on the environment and cellular state (**Table 1**). KLF4 regulates immune cell differentiation, polarization, and inflammatory responses, contributing to both effective pathogen defence and the prevention of excessive inflammation. Targeting KLF4 could offer new strategies for therapeutic interventions in inflammatory diseases and cancer. Its dual nature as a tumour suppressor and oncogene suggests that its therapeutic use may depend on the specific disease and context (**Figure 5**). In summary, KLF4 is a crucial regulator of immune cell function and disease progression, playing a key role in shaping the immune response and influencing the development and progression of various diseases.

**Table 1:** Summary of KLF4’s regulatory functions in immune cells and disease contexts.

Immune context	Cell type(s)	KLF4-Regulated Molecules/pathways	Regulatory effect	Associated disease(s)	References
Th17 Cell Differentiation	CD4+ T cells	IL-17, RORγt, STAT3	↑ Th17 differentiation	Autoimmune diseases (e.g., MS, RA)	(84, 85)
B Cell Maturation	B cells	Pax5, AID, Blimp-1	↑ Class switching and differentiation	Lymphomas, Autoimmune diseases	(86, 87)
Dendritic Cell Activation	Dendritic cells	IL-6, CD80, CD86	↑ Antigen presentation, T cell priming	Chronic infections, Cancer	(79, 88)
NK Cell Function	Natural Killer (NK) cells	IFN-γ, THBS1, CD47, ALSH1L	↑ Cytotoxicity and cytokine production	Viral infections, Tumors	(79, 82)
Cancer	Tumor-associated macrophages (TAMs), T cells	VEGF, PD-L1, NF-κB	↑ Tumor-promoting immune responses	Colon, Breast, and Lung cancers	(89, 90)
Inflammatory Bowel Disease (IBD)	Macrophages, T cells	IL-6, IL-12, STAT3	↑ Pro-inflammatory cytokines	Crohn’s disease, Ulcerative colitis	(91)
Inflammation (general)	Macrophages, Monocytes	IL-6, TNF-α, CCL2, NF-κB	↑ Pro-inflammatory gene expression	Atherosclerosis, Sepsis	(92)
Tissue Repair & M2 Polarization	Macrophages	IL-10, Arginase-1, STAT6	↑ Anti-inflammatory gene expression	Wound healing, Fibrosis	(93)
Atherosclerosis	Macrophages	ABCA1, ApoE, inflammatory cytokine	↑ Foam cell formation, ↑ inflammation	Cardiovascular disease	(92)
Pulmonary Fibrosis	Macrophages, Fibroblasts	TGF-β, MMPs	↑ Fibrogenic signaling	Idiopathic pulmonary fibrosis (IPF)	(94, 95)
Monocyte Differentiation	Monocytes	PU.1, CD14	↑ Monocyte differentiation from myeloid progenitors	Immune deficiencies, impaired myeloid development	(96)
Sepsis	Monocytes, Neutrophils	IL-1β, TNF-α, NF-κB	↑ Cytokine storm response	Systemic inflammatory response syndrome (SIRS)	(97)
Neutrophil Activation	Neutrophils	CD14, TLR4, IκBα kinase complex, IκBα, NF-κB, TNF-α	KLF4 deficiency: ↓ Neutrophil activation through TLR4—NF-κB signaling, ↓ CD14, ↓ IκBα phosphorylation, ↓ TNF-α	Experimental autoimmune encephalomyelitis (EAE), a model for multiple sclerosis (MS)	(45)

This table consolidates the regulatory functions of KLF4 across major immune cell types, including macrophages, T cells, B cells, dendritic cells, and NK cells. It highlights the cytokines, chemokines, and signalling pathways modulated by KLF4, delineating its dual role in promoting both pro- and anti-inflammatory responses depending on the cellular context and summarizes KLF4’s involvement in specific disease states, highlighting its relevance as a molecular target with diagnostic and therapeutic potential.

## 6 Conclusion

Work over the past two decades provides compelling evidence that KLF4 regulates key aspects of the innate and adaptive immune system. It orchestrates key processes across monocytes, macrophages, neutrophils, T cells, and B cells. In monocytes and macrophages, KLF4 acts downstream of PU.1 to promote lineage commitment and drives anti-inflammatory responses through M2-associated Stat6/IL-4 signaling while suppressing M1-associated NF-kB-mediated inflammation. In neutrophils, KLF4 regulates granule content, cytokine production, and bacterial killing by modulating the CD14/TLR4-NF-kB signaling pathway. In adaptive immunity, KLF4 inhibits the proliferation of CD8+ T cells and B cells through transcriptional repression of pro-survival and cell cycle-promoting genes such as NOTCH1 while also acting as a tumor suppressor.

Given the importance of the immune system in physiology (host defense) and disease (aging and age-associated disorders), efforts to target KLF4 may be therapeutically beneficial. In particular, given the intimate link between inflammation and aging (a.k.a. inflammaging), such effort may impact some diseases that constitute the largest source of morbidity, mortality, and healthcare expenditure worldwide.

Thus, future investigations should focus on the development of precision medicine to modulate KLF4 expression or function in a cell and disease-specific manner. Approaches may include RNA-based therapeutics or targeted epigenetic modifications, which will be critical for translating the immunoregulatory potential of KLF4 into clinically relevant therapies. Additionally, to clarify KLF4’s role in human inflammation and aging, emerging single-cell technologies such as single-cell multiomics and spatial transcriptomics can reveal its immune functions across diverse tissue and disease contexts.

Understanding the molecular mechanisms by which KLF4 modulates immune cell fate and function not only enhances our grasp of innate immunity but also opens promising avenues for therapeutic intervention in inflammatory diseases, autoimmune disorders, and tissue repair strategies. As research continues to unravel the complexity of transcriptional networks in immune cells, KLF4 stands out as a pivotal node with significant clinical relevance.
